# Multiple Pesticides and their Mixtures Tested for Genotoxicity in the Micronucleus Assays on Intestinal Caco-2 Cells

**DOI:** 10.5334/aogh.5345

**Published:** 2026-07-17

**Authors:** Francesca Truzzi, Eva Tibaldi, Roberta Noferini, Daria Sgargi, Simona Panzacchi, Gizem Nardali, Antonello Lorenzini, Silvia Dilloo, Eros D’Amen, Federica Gnudi, Giovanni Dinelli, Paul T. J. Scheepers, Daniele Mandrioli

**Affiliations:** 1 Department of Agricultural and Food Sciences, Alma Mater Studiorum-University of Bologna, Bologna, Italy; 2 Cesare Maltoni Cancer Research Center, Ramazzini Institute, Via Saliceto, 3, Bentivoglio, Bologna 40010, Italy; 3 Department of Biomedical and Neuromotor Sciences (DIBINEM), Alma Mater Studiorum-University of Bologna, Bologna, Italy; 4 Radboud Institute for Biological and Environmental Sciences, Radboud University, Nijmegen, Netherlands

**Keywords:** risk assessment, pesticides, Caco-2 cells, micronuclei, genotoxic

## Abstract

*Background:* Widespread exposure to multiple pesticides might potentially represent a genotoxic risk to humans. However, the effects of these mixtures are largely unknown. Genotoxicity is a key characteristic of carcinogens, and its assessment represents an important component of the safety assessment of pesticides.

*Methods:* In the present study, an *in vitro* micronucleus test on intestinal Caco-2 human cells was performed according to OECD TG 487. Ten pesticides were tested (dose range 0–100 mg L^–1^) either individually or as mixtures.

*Objectives:* Assessing the genotoxicity of 10 commonly used pesticides and their mixtures.

*Findings:* Significant dose-related increases in micronuclei were observed following exposure to lambda-cyhalothrin, tebuconazole, glyphosate, deltamethrin, fluopyram, and the synergist piperonyl butoxide. Significant increases in micronuclei were also observed at different doses for cypermethrin, acetamiprid, and cyprodinil; however, these increases were not dose dependent. Imazalil genotoxicity could not be analyzed due to the confounding effect of high cytotoxicity even at low doses. Results show that the co-formulant piperonyl butoxide was genotoxic to human cell lines at all tested doses. Moreover, glyphosate, acetamiprid, and fluopyram showed genotoxic effects at concentrations of 0.01–1.0 mg L^–1^. Although previously reported to be not genotoxic, cyprodinil and deltamethrin were observed to be genotoxic to Caco-2 cells. A combination of three prioritized pesticides (acetamiprid, glyphosate, tebuconazole) showed genotoxic effects even at the lowest dose. A combination of eight prioritized pesticides showed genotoxicity at the highest dose. No synergistic interactions in micronuclei formation were evident in either the mixture of three or eight prioritized pesticides.

*Conclusions:* This study provides important information on the genotoxicity of different widely used pesticides and confirms the validity of a component-based approach in the genotoxicity assessment of pesticide mixtures. This study was performed as part of the EU SPRINT (Sustainable Plant Protection Transition: A Global Health Approach) project.

## Introduction

Pesticides are chemical constituents commonly used in farming to prevent or control pests, including insects, rodents, fungi, weeds, and other unwanted organisms [1]. Global pesticide demand has increased substantially over time [2], and the intensive and widespread use of pesticides raises serious environmental and human health concerns [1, 3]. As part of the EU Horizon 2020 funded research project, SPRINT (Sustainable Plant Protection Transition: A Global Health Approach), 209 pesticide residues were examined in study sites of 10 separate European countries and 1 in Argentina that included conventional and organic farms. Mixtures of pesticide residues were shown to be omnipresent in agricultural environments (soil, water, sediment, crops, outdoor air) and in farmers’ households (indoor dust samples) [3]. Interestingly, measured concentrations of pesticide residues in soil were also shown to exceed predicted environmental residue concentrations, evidencing that current calculation methods may not reliably estimate the presence of pesticide residues [4]. To date, little is known about health risks posed by these environmentally relevant pesticide mixtures [3].

Acute and chronic health effects of pesticides are serious public health concerns, and within the European context, an urgent need to reduce pesticide use and risk was highlighted by the EU Commission and the European Environment Agency (EEA) due to widespread human exposure [5]. One of the potential chronic side effects of pesticides is genotoxicity. The strong link between DNA alterations and cancer or chronic degenerative diseases has long been known and widely reported [6–8]. The general terms “genotoxic” and “genotoxicity” apply to chemical agents which alter the structure, information content, or segregation of DNA, including those which cause DNA damage by interfering with normal replication processes [9]. Hence, genotoxicity assessment represents an important component of the overall safety assessment of pesticides, and further investigations are warranted [2]. Genotoxicity is one of the 10 key characteristics of carcinogens considered in IARC hazard assessment. The use of genotoxicity biomarkers, including chromosomal aberrations, micronuclei, sister chromatid exchanges, and comets, is considered suitable biomarkers for evaluating genotoxicity in cells in *in vitro* testing [10]. Among the methods to test for genotoxicity, the *in vitro* micronucleus assay has gained widespread international regulatory acceptance (OECD [Organization for Economic Co-operation and Development] Test Guideline No. 487) and is currently recommended in several authoritative documents [2, 11, 12]. The principle behind the assay is the measurement of micronuclei, which are formed during mitosis because of DNA damage from exposure to a test chemical. The cytokinesis-block version (prevention of cytoplasmic division into daughter cells) of the micronucleus assay is performed by using cytochalasin B (CytoB), which ensures that binucleate cells analyzed have undergone cell division, necessary to ascertain DNA damage and micronucleus formation. Given that the scoring of micronuclei is rendered inaccurate in the presence of cytotoxic doses of pesticides, the measurement of cytotoxicity is a requirement of the OECD Test Guideline No. 487 to ensure that genotoxic assessments are not confounded by cytotoxicity at the applied doses [12].

It is necessary to increase scientific evidence regarding the genotoxicity effects of many pesticides [10], as there is a lack of such data and often these are provided by the producers, rather than by independent laboratories [13]. The objective of the present research was to provide genotoxicity assessments of the prioritized (Top 10) pesticides and mixtures of greatest concern based upon SPRINT CSS exposure data. In particular, a quantitative ranking, based on weighted hazard quotient (wHQ) based on SPRINT CSS exposure data and available toxicity data, prioritized the following pesticides: lambda-cyhalothrin (insecticide), cypermethrin (insecticide), deltamethrin (insecticide), tebuconazole (fungicide), glyphosate (herbicide), acetamiprid (insecticide), cyprodinil (fungicide), piperonyl butoxide (synergist and co-formulant in pyrethroid-based pesticides), fluopyram (fungicide), and imazalil (fungicide). The pesticides were investigated individually as well as in mixtures, namely the Top 3 (acetamiprid + glyphosate + tebuconazole) and the Top 8 (acetamiprid + cypermethrin + cyprodinil + deltamethrin + glyphosate + lambda-cyhalothrin + piperonyl butoxide + tebuconazole), using the international regulatory accepted *in vitro* micronucleus assay on the human intestinal epithelial cell line, Caco-2. Notably, the genotoxicity of glyphosate has been widely debated [3, 14, 15], while cyprodinil, deltamethrin, fluopyram, and piperonyl butoxide have been generally considered non-genotoxic and scarcely studied in human cells [16–22]. Furthermore, it was also considered important to extend the dose range to include lower concentrations, even below those expected from exposure to ADI values of the respective pesticides [23, 24], which to date have largely been overlooked in published literature. For this reason, the comparative range selected was from 0.01 mg L^–1^ to 100 mg L^–1^ for each of the pesticides investigated.

## Materials and Methods

Micronucleus assay was performed according to OECD [Organization for Economic Co-operation and Development] Test Guideline No. 487 [12].

### Materials

The human epithelial colorectal adenocarcinoma Caco-2 cell line (ATCC® HTB-37TM) was purchased from distributors in Italy (Sesto San Giovanni, Milan, Italy). Dulbecco’s Modified Eagle Medium (DMEM), fetal bovine serum (FBS), and penicillin-streptomycin (Pen/Strep) were obtained from Gibco (Waltham, MA, USA). Cytochalasin B (CytoB) was from ACROS Organics (Segrate, MI, Italy), Vectashield Vibrance Antifade Mounting Medium with 4’,6-diamidino-2-phenylindole (DAPI) was purchased from Vector Laboratories (Newark, CA, USA). Imazalil (1-[2-(Allyloxy)-2-(2,4-dichlorophenyl) ethyl] imidazole) (>99% pure), fluopyram (N-[2-[3-Chloro-5-(trifluoromethyl)-2-pyridinyl]ethyl]-2-(trifluoromethyl) benzamide) (>98% pure), piperonyl butoxide (2-(2-Butoxyethoxy) ethyl (6-propylpiperonyl) ether) (>98% pure), cyprodinil (4-Cyclopropyl-6-methyl-N-phenylpyrimidin-2-amine) (>99% pure), Acetamiprid (N-(6-Chloro-3-pyridylmethyl)-N-cyano-N-methylacetamidine) (>99% pure), glyphosate (N-(Phosphonomethyl) glycine) (>98% pure), tebuconazole (N-(1-(4-Chlorophenyl)-4,4-dimethyl-3-(1H-1,2,4-triazol-1-ylmethyl)-3-pentanol) (>99% pure), deltamethrin (S)-Cyano(3-phenoxyphenyl) methyl (1R,3R)-3-(2,2-dibromoethen-1-yl)-2,2-dimethylcyclopropane-1-carboxylate) (>99% pure), cypermethrin ([Cyano-(3-phenoxyphenyl)methyl]3-(2,2-dichloroethenyl)-2,2-dimethylcyclopropane-1-carboxylate) (>99% pure) and lambda-cyhalothrin (RS)-alpha-cyano-3-phenoxybenzyl 3-(2-chloro-3,3,3-trifluoropropenyl)-2,2-dimethylcyclopropanecarboxylate) (>99% pure) were purchased from Merck Life Science S.r.l. (Milan, Italy). All remaining reagents used in the experiments were of analytical grade.

### Cell line and culture conditions

The Caco-2 human cell line was used. Cells were cultured in DMEM with 10% FBS and 1% Pen/Strep at 37°C in a humidified incubator with 5% CO_2_ in tissue culture flasks (75 cm^2^; BD Biosciences, Franklin Lakes, NJ, USA). The medium was changed every two days. Prior to experimentation, the cells were trypsinized to enable distribution of equivalent cell seeding densities to wells and cell density evaluated microscopically using a Bürker counting chamber.

### Preparation of the individual and combination pesticide treatments

The following 10 pesticides were investigated as mono-constituents: acetamiprid, cypermethrin, cyprodinil, deltamethrin, fluopyram, glyphosate, imazalil, lambda-cyhalothrin, piperonyl butoxide, and tebuconazole. Two combination pesticide treatments were used, namely the Top 3 (acetamiprid + glyphosate + tebuconazole) and the Top 8 (acetamiprid + cypermethrin + cyprodinil + deltamethrin + glyphosate + lambda-cyhalothrin + piperonyl butoxide + tebuconazole), respectively.

For the individual pesticide treatments, each of the 10 pesticides (5 mg) was dry-mixed with DMEM powder (500 mg) using a ceramic pestle and a mortar. For each pesticide, two separate stock solutions of 100 mg L^–1^ and 10 mg L^–1^ were prepared by diluting the powdered mixtures in distilled water. The solution remained clear, with no visible precipitate. The 100 mg/L solution was sonicated for 5 min. Prior to use on the cell cultures, all stock solutions were filtered using 0.45-μm diameter polyethersulfone (PES) filters.

For the Top 3, each of acetamiprid, tebuconazole, and glyphosate was dry-mixed with DMEM powder (1000 mg) using a ceramic pestle and a mortar. A stock solution of 100 mg L^–1^ was prepared following dilution in distilled water. The solution was sonicated for 5 min and filtered with a 0.45-μm diameter PES filter. NaCO_3_ (50 μL 10 mL^–1^) was then added to the solution. Prior to preparing the 10 mg L^–1^ stock solution, three separate 30 mg L^–1^ stock solutions were prepared by dry-mixing each pesticide (3 mg) with DMEM powder (1 mg) and adding distilled water. The three 30 mg L^–1^ stock solutions were sonicated for 5 min, filtered with 0.45-μm diameter PES filters. NaCO_3_ (50 μL 10 mL^–1^) was added to each solution. Equimolar concentrations of each of the three 30 mg L^–1^ stocks were then combined to produce the 10 mg L^–1^ solution of each pesticide. Similarly, for the Top 8, acetamiprid, cypermethrin, cyprodinil, deltamethrin, glyphosate, lambda-cyhalothrin, piperonyl butoxide, and tebuconazole (10 mg each), were dry-mixed with DMEM (1000 mg) and diluted in distilled water to attain a concentration of 100 mg L^–1^. The solution was sonicated for 5 min and then filtered through a 0.45-μm diameter PES filter before the addition of NaCO_3_ (50 μL 10 mL^–1^). As with the Top 3, 80 mg L^–1^ solutions were, similarly, prepared by dry-mixing each pesticide (8 mg) separately with DMEM powder (1 g). The same concentrations of each 80 mg/L pesticide solution were combined to produce the final 10 mg L^–1^ solution.

The 10 mg L^–1^ stock solutions for both the mono-constituent, as well as the Top 3 and Top 8 combination treatments, were subsequently diluted in liquid DMEM to produce concentrations of 0.01 mg L^–1^, 0.1 mg L^–1^, and 1 mg L^–1^, respectively.

### Culture conditions and administration of pesticide treatments to Caco-2 cells for the measurement of micronuclei

Caco-2 cells were plated in 4-well-chamberslides (3×10^4^ cells in 500 μL well^–1^) in Caco-2 medium (DMEM containing 10% FBS and 1% Pen/Strep) and incubated for 24 h to allow cell attachment and recovery prior to treatment. Cell seeding density had been optimized in preliminary experiments to ensure suitable cell growth throughout the assay and reliable micronucleus scoring. Throughout the procedure, all incubation periods were performed at 37°C in a humidified incubator with 5% CO_2_. The Caco-2 medium was removed and replaced with pesticide treatments (10 mono-constituent treatments and 2 combinations) at concentrations of 0.01 mg L^–1^, 0.1 mg L^–1^, 1.0 mg L^–1^, 10 mg L^–1^, and 100 mg L^–1^, respectively. The negative untreated control (CTRL) contained only DMEM. The experimental exposure period was 4 h. Thereafter, the medium containing the treatments was removed and replaced with fresh Caco-2 medium. The plates were incubated for a further 48 h, after which 4 μg mL^–1^ DMEM CytoB was added to each treatment well for 24 h. Thereafter, the medium was aspirated, and the cells washed with 1 x PBS (500 μL well^–1^), followed by 75 mM KCl (500 μL well^–1^) for 2 min at room temperature. The KCl was then removed and plates washed again with 1 x PBS (500 μL well^–1^). Cells were subsequently fixed in a solution of methanol: glacial acetic acid (3:1) and incubated for 10 min at room temperature. The solution was then removed. After removing the chamber side walls, the cell monolayers were either stained immediately for use or maintained at −20°C until a suitable time.

### Staining and microscopic viewing of micronuclei in Caco-2 cells

Unstained slides maintained at −20°C were removed and allowed to thaw at room temperature for 10 min. Thereafter, slides were incubated in PBS in a vertical position for 10 min, then placed in a horizontal position and stained. Staining was performed by adding Vectashield Vibrance Antifade Mounting Medium with DAPI. Since each slide contained four treatment wells, one drop of DAPI was added to each treatment. A cover slip was placed over the slide and sealed with transparent nail polish. Slides were viewed using a fluorescent Nikon Ni-E microscope (Nikon, Japan). To take photos, the image software used was NIS ELEMENTS AR Version 5.30.06 64 bit (Nikon, Japan). For imaging, the monochromatic camera (Qi2) was first chosen to activate the program. Thereafter, the blue filter for DAPI staining was selected with the selected Eyepiece-EPI to view the cells at a magnification of x 40. After determining the location of the cells, the magnification objective was changed to x 60 magnification and immersion oil added. The cells were viewed from the computer by replacing the Eyepiece-EPI selection to Qi2-EPI. A total of 40 photos were taken for each slide (10 photos well^–^^1^) at random positions, and each photo saved in jpeg format for analysis and elaboration of digital images.

### Photographic scoring criteria of Caco-2 cells for the CBPI index and the presence of micronuclei

Since CytoB was used, estimates of cytotoxicity were based on the Cytokinesis-Block Proliferation Index (CBPI). The CBPI scoring procedure includes the number of mononucleated, binucleated, and polynucleated cells, respectively, as well as the total number of cells. Additionally, at least 1000 binucleated cells/culture were examined for the presence of micronuclei. The number of binucleated cells in the positive CTRLs and treated groups subject to high pesticide dose (high toxicity index) may be <1000 cells. Binucleated cells were scored based on the following criteria: the presence of nuclei that were separate and of equal size, the presence of nuclei that may be in contact but with distinguishable nuclear boundaries, and nuclei that were linked by nucleoplasmic bridges. Exclusion criteria included cells in which the main nuclei were undergoing apoptosis (micronuclei may have already disappeared due to apoptosis). The inclusion of mononucleates in the calculation accounts for cytostasis and as such CBPI provides an estimate of cell proliferation. CBPI for the pesticide-treated cells and CTRLs were calculated separately.

The following formula was used:


$$C\,B\,P\,I=\frac{\begin{array}{c} N\,u\,m\,b\,e\,r\;o\,f\;m\,o\,n\,o\,n\,u\,c\,l\,e\,a\,t\,e\;c\,e\,l\,l\,s+2\times N\,u\,m\,b\,e\,r\;o\,f\;b\,i\,n\,u\,c\,l\,e\,a\,t\,e\;c\,e\,l\,l\,s\\ +3\times N\,u\,m\,b\,e\,r\;o\,f\;m\,u\,l\,t\,i\,n\,u\,c\,l\,e\,a\,t\,e\;c\,e\,l\,l\,s \end{array}}{T\,o\,t\,a\,l\;n\,u\,m\,b\,e\,r\;o\,f\;c\,e\,l\,l\,s}$$

The cytotoxicity estimate was calculated as follows:


$$Cytotoxicity=100-100*\frac{CBPI_{T-1}}{CBPI_{C-1}}$$

where *T* = test pesticide treated cultures, *C* = CTRL cultures.

The criteria for scoring micronuclei were as follows: diameters not exceeding one-third of the main nucleus, the presence of micronuclei separate from the main nucleus, the presence of micronuclei that marginally overlap with the main nucleus but with distinguishable nuclear boundaries, a similar staining pattern between micronuclei and the main nucleus, and finally that micronuclei be located near the edge of the main nucleus of the binucleated cell.

### Analysis of images

Digital images were analyzed using FIJI Image J-win 64 which was downloaded free of charge. The jpeg images were opened and the following procedure sequence followed: Plugins–Analyzed–Cell Counter–Initialized. The types of cells that were counted included mononucleated, binucleated, and polynucleated cells, respectively, as well as those containing micronuclei. The software recorded the total number of each cell type counted. Numbers were assigned to distinguish the cell types as follows: (1) binucleated cells, (2) polynucleated cells, (3) mononucleated cells, and (4) micronuclei. The data obtained from the counting with the Image J software were transferred to an Excel file.

### Statistical analysis

Mononucleated, binucleated, multinucleated cells, and the number of micronuclei were assessed for samples from all groups. The number of micronuclei on the total of binucleated cells was compared between the controls (treated with CytoB) and all doses of each treatment using a one-sided Fisher Exact test. A concurrent negative control was included with each pesticide experiment and served as the corresponding reference group for statistical comparisons. In accordance with OECD Test Guideline No. 487, assay validity was established through the evaluation of the recommended number of binucleated cells and in compliance with the guideline acceptance criteria. Furthermore, the presence of a linear trend across doses was verified using a Cochran–Armitage trend test. Results were considered statistically significant when the *P*-values were <0.05. Statistical analyses were performed using the German Cancer Research Center software (https://biostatistics.dkfz.de/) and Stata 18 software (StataCorp 2023. Stata Statistical Software: Release 18. College Station, TX: StataCorp LLC).

### Validation of the efficacy of the negative and positive controls for the *in vitro* micronucleus test

In the present study, the negative CTRL, treated with CytoB only, was first tested to confirm the formation of binucleate cells and to provide the basal estimate of micronuclei in a minimum number of 1000 binucleate cells. A total of 34 micronuclei were counted for a total of 1037 Caco-2 binucleate cells (Table 1). Then, Mito C, known to induce chromosomal breakage, was the selected clastogenic positive CTRL. Efficacy of the positive CTRL was verified by the formation of 217 micronuclei in 1009 Caco-2 binucleate cells (Table 1). Neither CytoB nor Mito C was shown to be cytotoxic to the Caco-2 cells at that concentration. Cytotoxicity ranged from 0.96% to 1.90% (Table 1). These values were significantly below the 55.0 ± 5.0% set target, indicating that the administration of the positive and negative CTRLs did not interfere with the scoring of micronuclei due to cytotoxic effects on the cells.

**Table 1:** The effects of the non-pesticide containing negative control (CTRL) with Cytochalasin B (CytoB) and positive CTRL with clastogenic mitomycin C (Mito C) on the number of binucleated cells and genotoxic micronuclei as well as the cytotoxicity percentages in Caco-2 cells.

	PESTICIDE DOSE	BINUCLEATED CELLS (NUMBER)	MICRONUCLEI (NUMBER)	SIGNIFICANT INCREASE	CYTOTOXICITY (%)
CTRL (only CytoB)	0	1027	34		0.96
Mito C	0	1009	217	*	1.90

*1-sided Fisher Exact test (*P* < 0.01).

## Results

**Figure 1:**
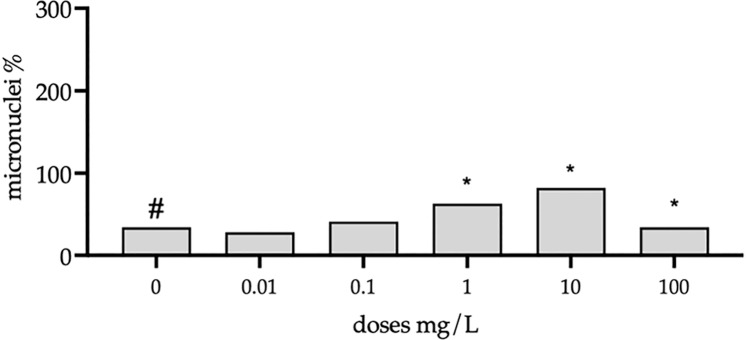
Effects of pure lambda-cyhalothrin, within the dose range 0–100 mg L^–1^, on the number of micronuclei per 1000 binucleate Caco-2 cells. Data for each dose is expressed as a single value. The presence of increased linear trend was evaluated using 1-sided Cochran–Armitage trend test (^#^*P* < 0.05). Pairwise comparisons of the incidence of micronuclei over binucleated cells were performed using the 1-sided Fisher’s exact test (*P < 0.05).

**Figure 2:**
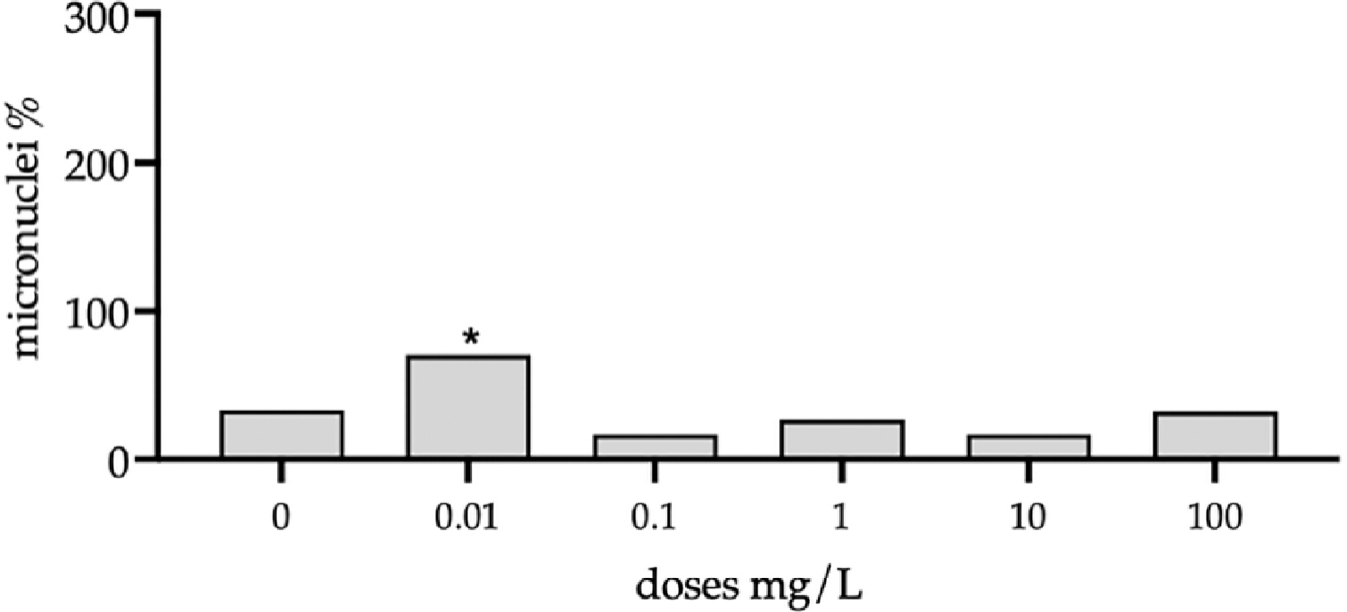
Effects of pure cypermethrin, within the dose range 0–100 mg L^–1^, on the number of micronuclei per 1000 binucleate Caco-2 cells. Data for each dose is expressed as a single value. The presence of increased linear trend was evaluated using 1-sided Cochran–Armitage trend test (^#^*P* < 0.05). Pairwise comparisons of the incidence of micronuclei over binucleated cells were performed using the 1-sided Fisher’s exact test (*P < 0.05).

**Figure 3:**
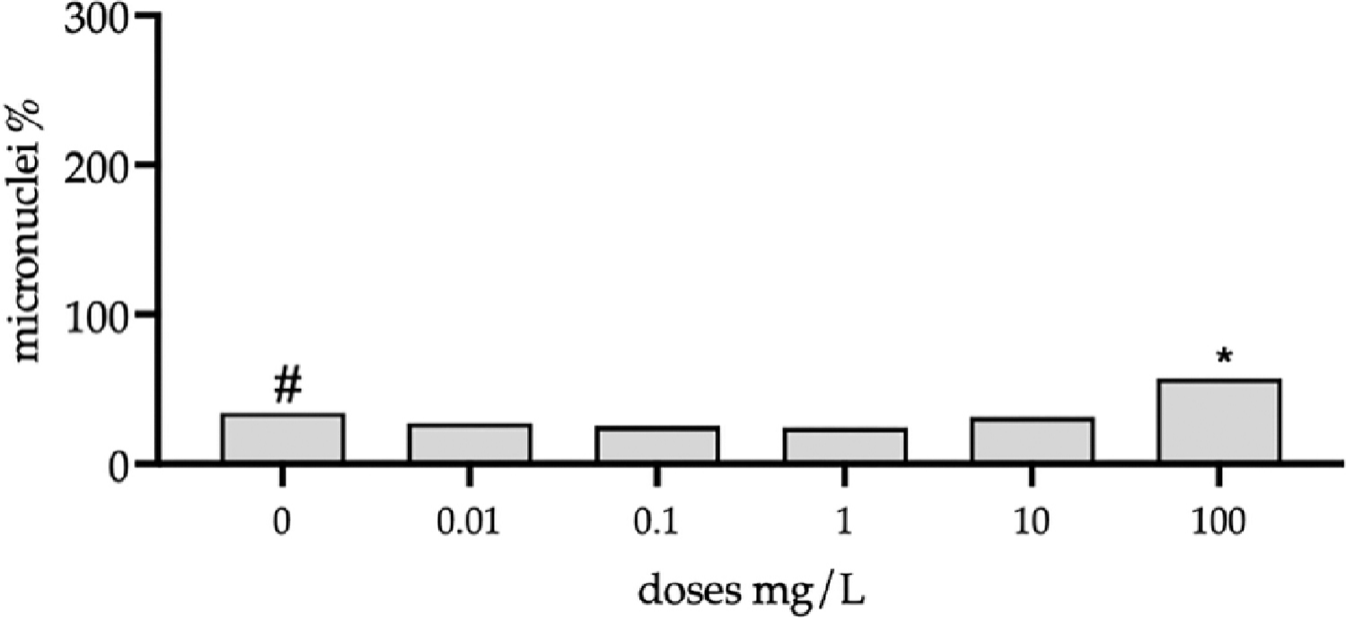
Effects of pure deltamethrin, within the dose range 0–100 mg L^–1^, on the number of micronuclei per 1000 binucleate Caco-2 cells. Data for each dose is expressed as a single value. The presence of increased linear trend was evaluated using 1-sided Cochran–Armitage trend test (^#^*P* < 0.05). Pairwise comparisons of the incidence of micronuclei over binucleated cells were performed using the 1-sided Fisher’s exact test (*P < 0.05).

**Figure 4:**
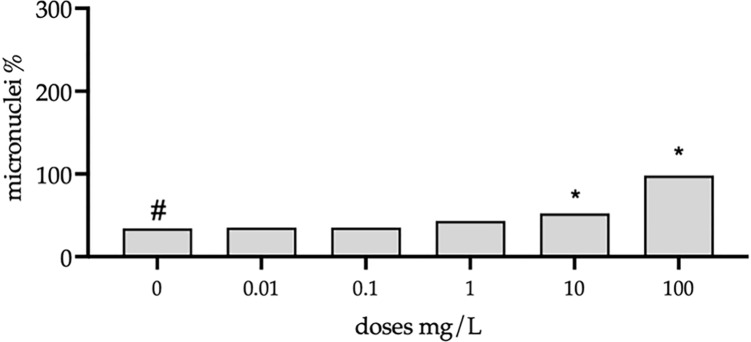
Effects of pure tebuconazole, within the dose range 0–100 mg L^–1^, on the number of micronuclei per 1000 binucleate Caco-2 cells. Data for each dose is expressed as a single value. The presence of increased linear trend was evaluated using 1-sided Cochran–Armitage trend test (^#^*P* < 0.05). Pairwise comparisons of the incidence of micronuclei over binucleated cells were performed using the 1-sided Fisher’s exact test (*P < 0.05).

**Figure 5:**
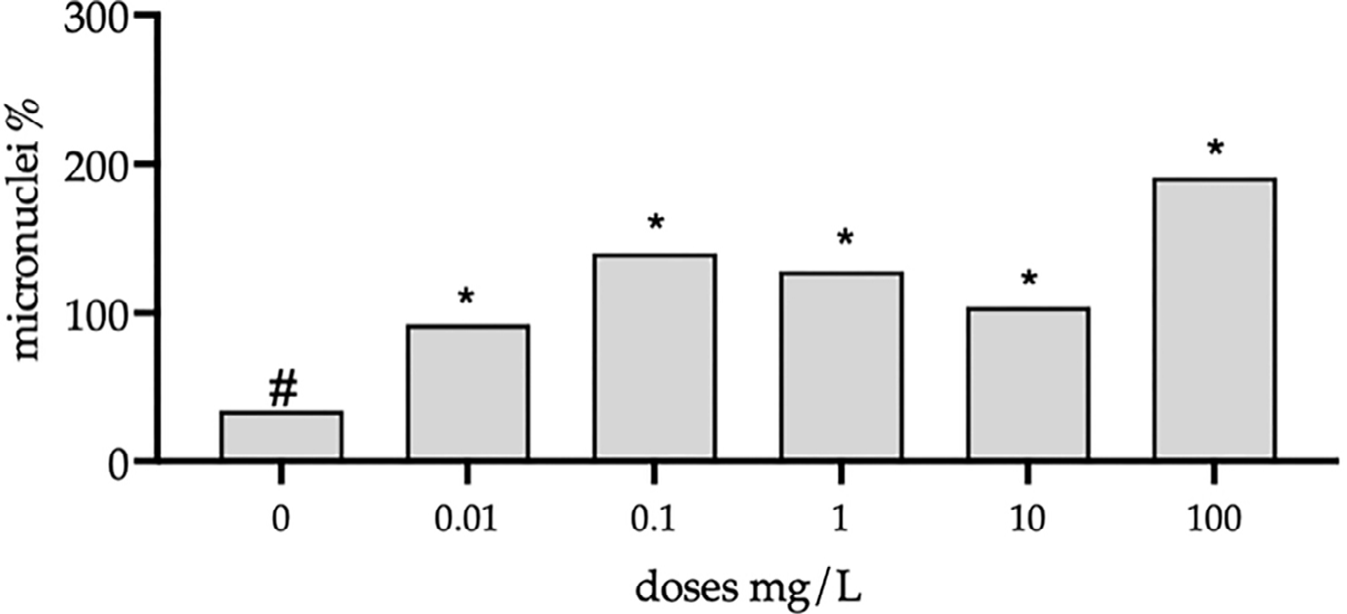
Effects of pure glyphosate, within the dose range 0–100 mg L^–1^, on the number of micronuclei per 1000 binucleate Caco-2 cells. Data for each dose is expressed as a single value. The presence of increased linear trend was evaluated using 1-sided Cochran–Armitage trend test (^#^*P* < 0.05). Pairwise comparisons of the incidence of micronuclei over binucleated cells were performed using the 1-sided Fisher’s exact test (*P < 0.05).

**Figure 6:**
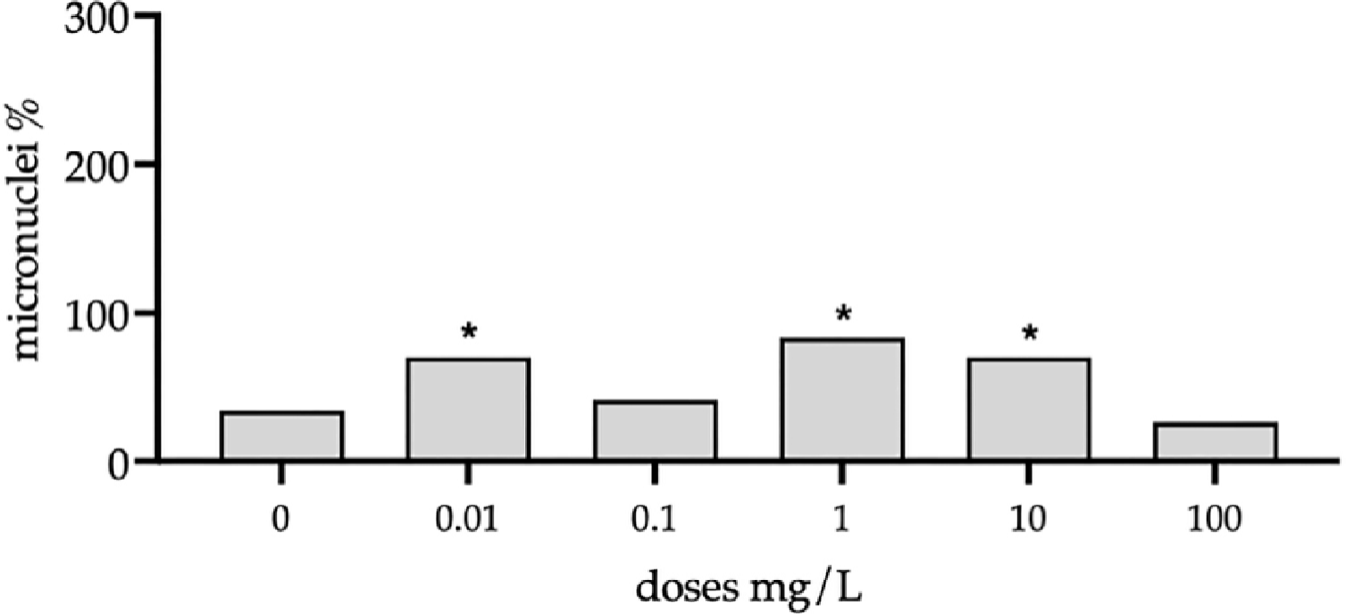
Effects of pure acetamiprid, within the dose range 0–100 mg L^–1^, on the number of micronuclei per 1000 binucleate Caco-2 cells. Data for each dose is expressed as a single value. The presence of increased linear trend was evaluated using 1-sided Cochran–Armitage trend test (^#^*P* < 0.05). Pairwise comparisons of the incidence of micronuclei over binucleated cells were performed using the 1-sided Fisher’s exact test (*P < 0.05).

**Figure 7:**
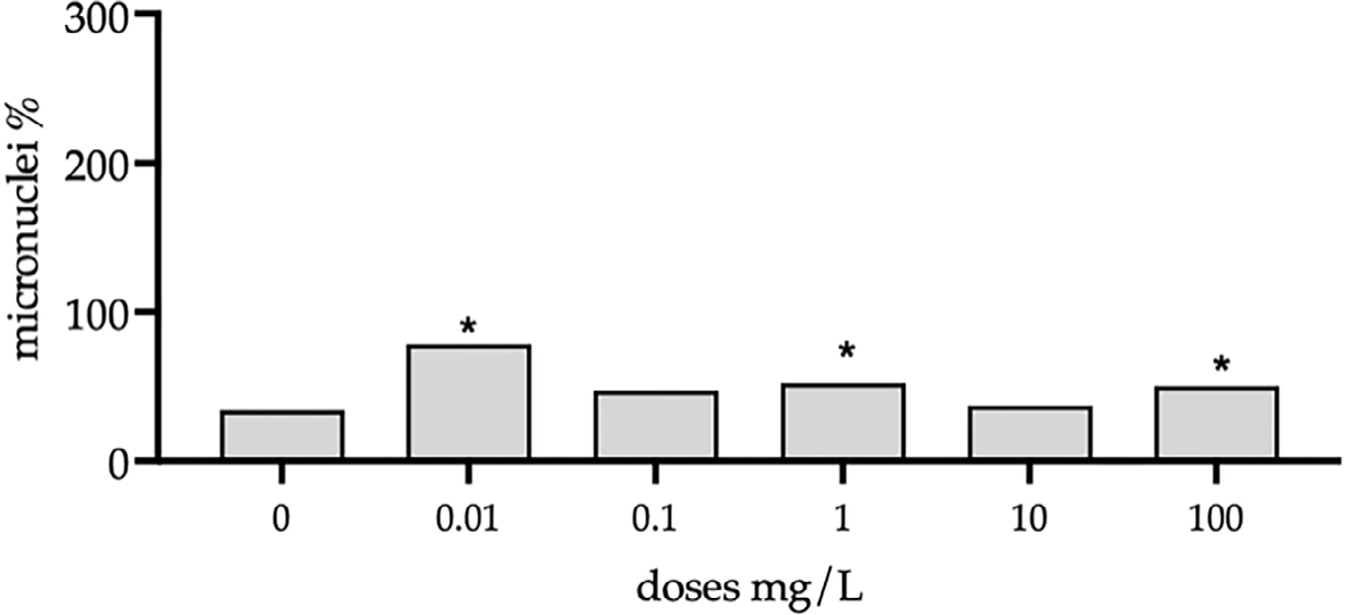
Effects of pure cyprodinil, within the dose range 0–100 mg L^–1^, on the number of micronuclei per 1000 binucleate Caco-2 cells. Data for each dose is expressed as a single value. The presence of increased linear trend was evaluated using 1-sided Cochran–Armitage trend test (^#^*P* < 0.05). Pairwise comparisons of the incidence of micronuclei over binucleated cells were performed using the 1-sided Fisher’s exact test (*P < 0.05).

**Figure 8:**
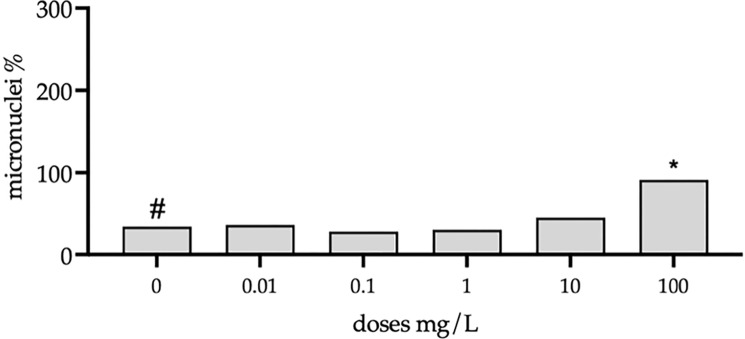
Effects of pure piperonyl butoxide, within the dose range 0–100 mg L^–1^, on the number of micronuclei per 1000 binucleate Caco-2 cells. Data for each dose is expressed as a single value. The presence of increased linear trend was evaluated using 1-sided Cochran–Armitage trend test (^#^*P* < 0.05). Pairwise comparisons of the incidence of micronuclei over binucleated cells were performed using the 1-sided Fisher’s exact test (*P < 0.05).

**Figure 9:**
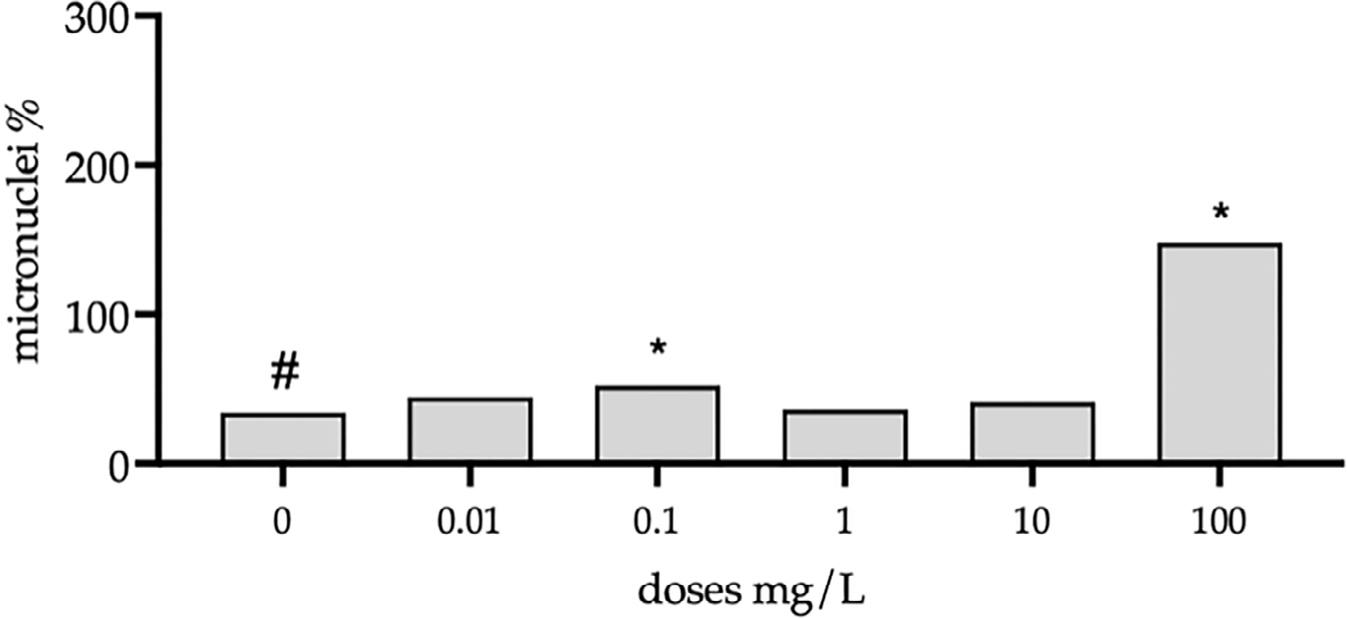
Effects of pure fluopyram, within the dose range 0–100 mg L^–1^, on the number of micronuclei per 1000 binucleate Caco-2 cells. Data for each dose is expressed as a single value. The presence of increased linear trend was evaluated using 1-sided Cochran–Armitage trend test (^#^*P* < 0.05). Pairwise comparisons of the incidence of micronuclei over binucleated cells were performed using the 1-sided Fisher’s exact test (*P < 0.05).

**Figure 10:**
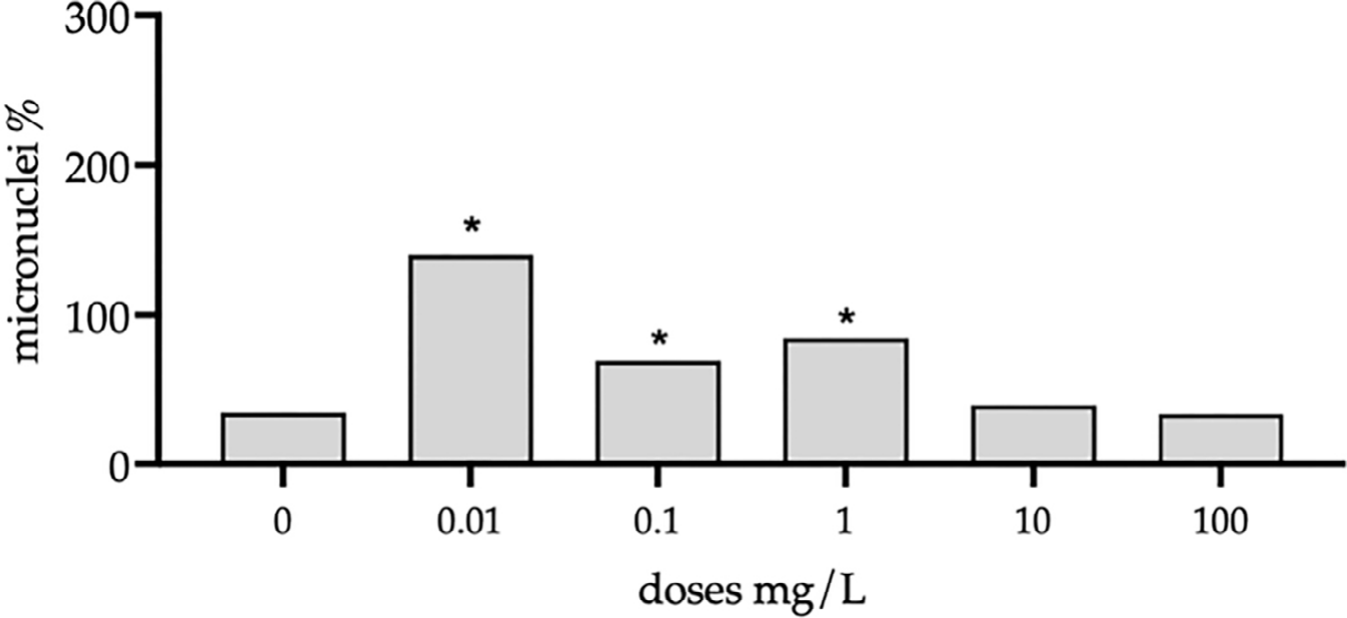
Effects of a combination mixture of pesticides called Top 3 (pure acetamiprid, tebuconazole, glyphosate), within the dose range 0–100 mg L^–1^, on the number of micronuclei per 1000 binucleate Caco-2 cells. Data for each dose is expressed as a single value. The presence of increased linear trend was evaluated using 1-sided Cochran–Armitage trend test (^#^*P* < 0.05). Pairwise comparisons of the incidence of micronuclei over binucleated cells were performed using the 1-sided Fisher’s exact test (*P < 0.05).

**Figure 11:**
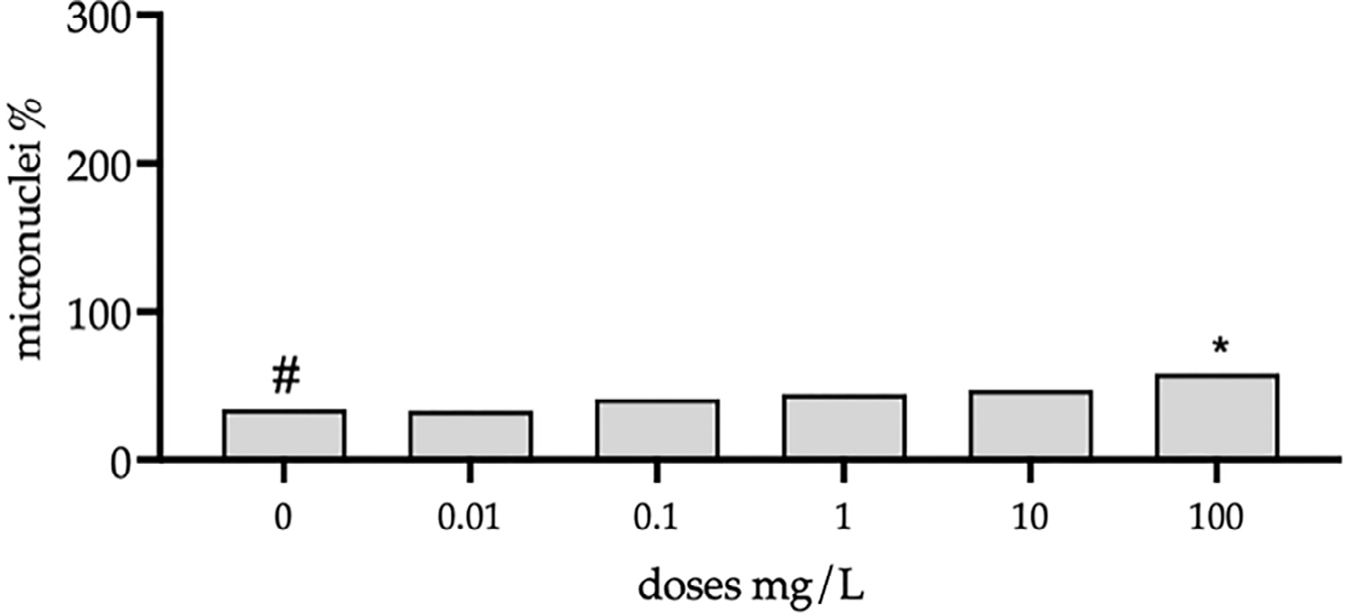
Effects of a combination mixture of pesticides called Top 8 (pure lambda-cyhalothrin, cypermethrin, deltamethrin, tebuconazole, glyphosate, acetamiprid, cyprodinil, piperonyl butoxide), within the dose range 0–100 mg L^–1^, on the number of micronuclei per 1000 binucleate Caco-2 cells. Data for each dose is expressed as a single value. The presence of increased linear trend was evaluated using 1-sided Cochran–Armitage trend test (^#^*P* < 0.05). Pairwise comparisons of the incidence of micronuclei over binucleated cells were performed using the 1-sided Fisher’s exact test (*P < 0.05).

The results of the micronuclei genotoxicity assays of each of the 10 single pesticides and the 2 mixtures tested at 5 dose levels are provided here. Pairwise comparisons of the incidence of micronuclei over binucleated cells were performed within the dose range of 0–100 mg L^–1^ for each of the pesticides as mono-constituents (Figures 1–9, Tables 2 and 3) and in the combination treatments (Figures 10 and 11). Significantly increasing concentrations of pesticides with the highest incidence of micronuclei present at the final dose of 100 mg L^–1^ were evident from exposure to lambda-cyhalothrin (Figure 1), deltamethrin (Figure 3), tebuconazole (Figure 4), glyphosate (Figure 5), piperonyl butoxide (Figure 8), fluopyram (Figure 9), and the Top 8 (Figure 11), respectively. The Cochran–Armitage trend test showed a significant dose-related increase for lambda-cyhalothrin, deltamethrin, tebuconazole, glyphosate, piperonyl butoxide, fluopyram, and the Top 8. A total of 273 micronuclei were counted at 100 mg L^–1^ glyphosate, which exceeded the number induced by the known clastogenic agent, mitomycin C (Figure 5, Table 1). At 100 mg L^–1^, the numbers of micronuclei for fluopyram were 150, with numbers declining close to 100 for tebuconazole and piperonyl butoxide before decreasing to approximately 50 micronuclei for deltamethrin, lambda-cyhalothrin, and Top 8, respectively.

**Table 2:** The effect of imazalil doses on binucleate cell number, micronuclei number, and cytotoxicity in Caco-2 cells.

DOSE (MG L^–1^)	BINUCLEATED CELLS (NUMBER)	MICRONUCLEI (NUMBER)	CYTOTOXICITY (%)
0	1027	34	
0.01	1008	14	38.82
0.1	1024	12	39.34
1	1010	13	38.66
10	1011	12	42.00
100	128	13	82.61

**Table 3:** Cytotoxicity assessments and micronuclei/binucleate cell number on Caco-2 cells for the pesticides, as mono-constituents, for all doses (0–100 mg L^–1^).

SUBSTANCE	DOSE	% CYTOTOXICITY	MICRONUCLEI/BINUCLEATE TOT X 1000
Control	0	**–**	33.11
Lambda-cyhalothrin	0.01	42.08	27.42
Lambda-cyhalothrin	0.1	39.43	40.35
Lambda-cyhalothrin	1	37.44	62.62
Lambda-cyhalothrin	10	39.91	80.23
Lambda-cyhalothrin	100	57.54	56.86
Cypermethrin	0.01	26.56	70.44
Cypermethrin	0.1	26.52	40.59
Cypermethrin	1	32.02	26.81
Cypermethrin	10	28.72	30.82
Cypermethrin	100	12.67	32.00
Deltamethrin	0.01	36.63	26.24
Deltamethrin	0.1	34.75	22.36
Deltamethrin	1	40.05	23.35
Deltamethrin	10	38.85	30.54
Deltamethrin	100	38.22	52.58
Tebuconazole	0.01	36.88	34.21
Tebuconazole	0.1	37.55	33.85
Tebuconazole	1	36.11	42.32
Tebuconazole	10	38.00	50.93
Tebuconazole	100	34.92	97.03
Glyphosate	0.01	24.17	90.91
Glyphosate	0.1	10.40	107.44
Glyphosate	1	11.71	97.78
Glyphosate	10	10.78	98.58
Glyphosate	100	22.98	268.44
Acetamiprid	0.01	19.78	68.63
Acetamiprid	0.1	27.47	40.96
Acetamiprid	1	18.92	79.81
Acetamiprid	10	20.00	68.76
Acetamiprid	100	31.72	25.69
Cypronidil	0.01	35.26	75.73
Cypronidil	0.1	38.62	46.72
Cypronidil	1	40.43	51.90
Cypronidil	10	42.88	36.96
Cypronidil	100	42.96	49.02
Piperonyl butoxide	0.01	36.78	35.79
Piperonyl butoxide	0.1	36.22	27.64
Piperonyl butoxide	1	34.54	29.97
Piperonyl butoxide	10	34.87	44.55
Piperonyl butoxide	100	36.25	88.61
Fluopyram	0.01	35.25	44.00
Fluopyram	0.1	34.48	49.52
Fluopyram	1	36.34	35.23
Fluopyram	10	38.99	39.84
Fluopyram	100	13.55	146.83

A significantly higher number of micronuclei was evident at all doses of glyphosate (Figure 5), showing that this pesticide was the most genotoxic to Caco-2 cells in comparison with the remaining mono-constituent and combination pesticides. Of interest, the presence of micronuclear doses in and around the ADI values (0.5 mg kg^–1^ body weight) was also evident for glyphosate (Figure 5).

The non-dose-related increases in micronuclei formation were characteristic of cypermethrin (Figure 2), acetamiprid (Figure 6), cyprodinil (Figure 7), and the Top 3 (Figure 10). For cypermethrin, cyprodinil, and the Top 3, respectively, the highest micronuclei formation occurred at the lowest concentration of 0.01 mg L^–1^ compared to all other doses. Micronuclei formation was only evident at 0.01 mg L^–1^ for cypermethrin (Figure 2), between 0.01 mg L^–1^ and 1 mg L^–1^ for the Top 3 (Figure 11), between 0.01 mg L^–1^ and 10 mg L^–1^ for acetamiprid (Figure 6), and between 0.01 mg L^–1^ and 100 mg L^–1^ for cyprodinil (Figure 7), respectively. These values coincide with those in the range around the respective ADI values.

Imazalil could not be effectively assessed for genotoxic potential based on cytotoxicity effects observed at the 100 mg L^–1^ dose, coinciding with a significant decrease in the number of binucleate cells (Table 2). Moreover, the number of micronuclei at all doses was shown to be inferior to the baseline number of micronuclei in the CytoB CTRL (Tables 1 and 2).

### Comparison of the combination pesticides in relation to the respective individual mono-constituents on micronuclei formation in binucleate Caco-2 cells

For the Top 3 (acetamiprid + glyphosate + tebuconazole), both glyphosate and tebuconazole demonstrated dose-related increases in micronuclei formation as mono-constituents while acetamiprid was non-dose-related (Figures 4–6). In combination, the significantly highest genotoxic effect at the lowest concentration at 0.01 mg L^–1^ appeared additive from the singular contributions of acetamiprid and glyphosate (Figure 10). No effect was evident at the 10–100 mg L^–1^ concentration range (Figure 10), even though the highest micronuclei formation was recorded at this range for both glyphosate and tebuconazole individually (Figures 4–5).

For the Top 8 (acetamiprid + cypermethrin + cyprodinil + deltamethrin + glyphosate + lambda-cyhalothrin + piperonyl butoxide + tebuconazole), individually, five pesticides displayed dose-related genotoxic increases (Figures 1, 3–5, 8), whereas three displayed non-dose-related increases (Figures 2, 6 and 7). In combination, only the 100 mg L^–1^ dose produced significantly higher numbers of micronuclei (Figure 11), which were equivalent in number to that shown individually for lambda-cyhalothrin (Figure 1), deltamethrin (Figure 3), and cyprodinil (Figure 7), respectively. No synergistic interactions were reported for either the Top 3 or the Top 8.

### Effects of the doses of the 10 pesticides and 2 combination treatments on the cytotoxicity in Caco-2 cells

For the cytotoxicity assessment, the CBPI was calculated for each pesticide dose, a requirement when evaluating genotoxic effects as outlined in the OECD Test Guideline No. 487 (OECD, 2023). A 55 ± 0.5% cytotoxicity target was provided (OECD 2023). Micronuclei frequencies can be significantly over or alternatively underestimated at doses that induce toxicity more than 55%, whereas all values below 55% are not considered cytotoxic. From the present results, the range of doses (0.01–100 mg L^–1^) used for all the pesticides analyzed were not cytotoxic to the accurate estimation of micronuclei (Tables 3 and 4). Only one outlier was observed in the 100 mg/L treatment with lambda-cyhalothrin, which raised the percentage to 57%, but was included in the analysis because it was still very close to the established threshold (OECD 2023). The most striking case was for Imazalil at 100 mg L^–1^ (Table 2) which was then not considered in the analysis.

**Table 4:** Cytotoxicity assessments on Caco-2 cells for the pesticides in combination for all doses (0–100 mg L^–1^).

SUBSTANCE	DOSE	% CYTOTOXICITY	MICRONUCLEI/BINUCLEATE TOT X 1000
Top 3	0.01	36.63	135.14
Top 3	0.1	39.72	66.86
Top 3	1	39.57	83.50
Top 3	10	37.41	38.77
Top 3	100	38.24	32.16
Top 8	0.01	40.54	32.07
Top 8	0.1	43.97	40.96
Top 8	1	44.53	43.39
Top 8	10	44.80	45.85
Top 8	100	42.10	57.43

## Discussion

Given the widespread public health concerns from the use of potentially genotoxic pesticides in the agricultural sector, the present research addressed the following critical points: (1) reducing the use of animals in risk assessments; (2) use of new *in vitro* approaches that can more efficiently test, classify, and prioritize chemicals for evaluation and regulation; (3) the use of human cells in *in vitro* approaches, more specifically the use of epithelial cells as a “site of first contact” for which there is demand; (4) strict adherence to internationally recognized protocols for comparative purposes when comparing research; (5) selection of suitable pesticide doses in which genotoxic assessments were not confounded by cytotoxicity of the applied doses, and (6) re-evaluation of known pesticides for which research on human cells was either lacking or produced inconsistent or confounding results.

In response to points (1) to (3) above, the present study employed an *in vitro* approach, specifically using human intestinal epithelial Caco-2 cells as the “site of first contact via oral ingestion” model system. To the best of our knowledge, only one study using Caco-2 cells reported the genotoxicity of acetamiprid, but cytotoxicity was also a confounding factor at certain doses in the assay system [25].

In line with points (4) and (5), the present study strictly adhered to the recommendations of the *in vitro* micronucleus test [12], which has gained increasingly widespread international regulatory acceptance for *in vitro* genotoxic testing. From the controls implemented (presence of the required number of binuclear cells at all doses, absence of interfering cytotoxicity at the selected pesticide doses, the presence of numerous micronuclei when using a known clastogenic agent), the efficacy of the *in vitro* micronucleus test procedure for Caco-2 cells was verified. This study addressed point (6) by reassessing the genotoxic effects in human cell lines of the 10 pesticides prioritized by the SPRINT project as being of concern for EU farmers and consumers.

Overall, research on genotoxic effects of known pesticides on human cells is scarce [11]. There are either no or insufficient studies using human cells for piperonyl butoxide, cyprodinil, deltamethrin, and fluopyram, which are largely considered non-genotoxic. Nonetheless, of particular interest is the considerable controversy regarding the genotoxicity of glyphosate [3, 14, 15], despite IARC in 2015 state that “there is strong evidence that glyphosate causes genotoxicity.” In the present study, glyphosate exposure was shown to induce the highest percentage formation of micronuclei compared to the remaining pesticides in mono-constituent form. Glyphosate was shown to be genotoxic to epithelial Caco-2 cells from 0.01 mg L^–1^ to 100 mg L^–1^ (ca 0.06–600 μM), contrasting with previous reports using human lymphocyte cells. In white blood cells, it was shown that glyphosate alone was only genotoxic at concentrations exceeding either 100 μM [3, 15, 26] or 200 μM [14] and 300 μM [27]. In TK6 cells, genotoxicity was only shown above 10 mM glyphosate [28]. To the best of our knowledge, this is the first report using human epithelial cells showing genotoxicity by glyphosate from a single exposure alone at low concentration ranges of 0.01–1.0 mg L^–1^. Given that life-time daily tolerance to glyphosate without appreciable health concerns is set at 0.1 and 0.5 mg/kg body weight for accepted operator exposure levels (AOEL) and consumers (ADI), respectively [23, 24], the study highlights potential genotoxic effects to human epithelial cells from a single exposure at levels that are inferior and equal to current AOEL and ADI values.

The present study showed for the first time that synergist and co-formulant piperonyl butoxide was genotoxic to human cells. Piperonyl butoxide was genotoxic to Caco-2 cells at 100 mg L^–1^, despite being previously considered a non-genotoxic compound [18]. More recently, the need to reassess the hazards of piperonyl butoxide was highlighted [20]. In the latter editorial, genotoxic effects on animals were mentioned with no studies on human cells being reported. Moreover, although well-recognized to be highly cytotoxic compound, the administration of cyprodinil to Caco-2 cells, at a concentration as low as 0.01 mg L^–1^, resulted in micronuclei formation from a single exposure, despite being considered non-genotoxic from previous studies conducted on white blood cells [17, 21]. As with glyphosate, the 0.03 mg kg^–1^ ADI of cyprodinil [23, 24] is equivalent to the value inducing genotoxic effects in Caco-2 from a single dose. Furthermore, micronuclei formation was shown for the first time in human cells following exposure to deltamethrin alone, despite being considered non-genotoxic in previous reports [16, 19, 29]. Present results corroborated the genotoxicity potential of fluopyram (0.1, 100 mg L^–1^) which was only recently shown to be genotoxic for the first time in blood lymphocytes, measured at concentrations exceeding 50 mg L^–1^ [30]. Lambda-cyhalothrin, cypermethrin, tebuconazole, and acetamiprid were, similarly, all genotoxic to human intestinal Caco-2 cells. Due to cytotoxicity induced by imizalil, this was the only pesticide that could not be effectively evaluated for genotoxicity in the present study.

Present results corroborated the presence of genotoxicity at doses within the range used in the present study on cell lines for lambda-cyhalothrin [31, 32], cypermethrin [33–35], tebuconazole [36], and acetamiprid [25, 37], respectively. However, for acetamiprid, micronuclei formation in Caco-2 cells was also observed between 0.01 mg L^–1^ and 10 mg L^–1^, a range either not investigated in previous studies [25, 37] or investigated but found to be negative for increase of micronuclei in white blood cells [38]. Similarly, micronuclei formation at 0.01 mg L^–1^ cypermethrin in the present study corroborated previous results for this compound at 0.025 mg L^–1^ [35], highlighting the importance of administering pesticides at ADI levels. Previously, cypermethrin was only shown to be genotoxic at higher concentrations, although the 0.01–5 mg L^–1^ range was not investigated [33, 34].

For all the pesticides analyzed in mono-constituent form, two response mechanisms were evident, namely a dose-dependent increase in micronuclei formation and a non-dose-dependent incidence of micronuclei. Regarding the dose-dependent increases between 0 mg L^–1^ and 100 mg L^–1^, lambda-cyhalothrin, deltamethrin, tebuconazole, glyphosate, piperonyl butoxide, and fluopyram displayed a dose-dependent increase in micronuclei formation in Caco-2 cells. Previous works demonstrating dose-dependent increases within regions of the concentration ranges examined were evident for lambda-cyhalothrin [31], tebuconazole [36], glyphosate [26, 27], and fluopyram [30]. Regarding non-dose-dependent responses, cypermethrin, acetamiprid, and cyprodinil displayed non-dose-dependent incidences in micronuclei. It is important to emphasize that in some cases with clear linear trends, there is no doubt regarding the effect of pesticides on cells (fluopyram, piperonyl butoxide, glyphosate, tebuconazole, lambda-cyhalothrin, deltamethrin), whereas for cases where a single dose is statistically significant, further analysis is needed and the data must be replicated. Notably, we used a conservative model (Caco-2 cells) without metabolic activation, which might have enhanced the micronuclei response.

For the first time, the present study also addressed pesticides or mixtures that have either not yet been tested, or for which there is no previously reported evidence of adequate testing on human cell lines. For certain pesticides, as in the case of glyphosate, our results confirm similar previous findings. However, for the ones that have been reported here for the first time as genotoxic, further studies are recommended to confirm our results. Synergistic effects of pesticides in combination have been widely reported in the literature, and it was considered important to investigate this possibility. However, no synergistic interactions in micronuclei formation were evident in either the Top 3 or Top 8. Of relevance is that in the combination of the Top 3 (acetamiprid + glyphosate + tebuconazole), interactions between the respective constituents resulted in significant micronuclei formation at the lowest doses, not evident for either glyphosate or tebuconazole singularly, which were dose dependent. To the best of our knowledge, there are no reports of acetamiprid + glyphosate + tebuconazole combinations in the available literature presenting results that can be compared to the present results. For the Top 8, genotoxicity was evident at the highest dose, presumably because five of the eight pesticide constituents demonstrated dose-dependent increases in micronuclei.

## Conclusions

In conclusion, the present study showed that lambda-cyhalothrin, cypermethrin, deltamethrin, tebuconazole, glyphosate, cyprodinil, piperonyl butoxide, fluopyram, and two mixtures of three and of eight compounds were genotoxic to intestinal epithelial Caco-2 cells. Dose-dependent responses in micronuclei formation were evident for lambda-cyhalothrin, tebuconazole, glyphosate, deltamethrin, fluopyram, and piperonyl butoxide. Instead, non-dose-dependent increases in micronuclei were evident for cypermethrin, acetamiprid, and cyprodinil. Glyphosate, by single testing and combined with acetamiprid and fluopyram, showed significant micronuclei increases in intestinal cells at lower doses (0.01–0.1 mg L^–1^) than those reported previously for human white blood cells. These results highlighted the importance of extending the tested dose ranges to include ADI range values. Like the results shown previously for cypermethrin on blood lymphocytes and a HepG2 cell line [35], the present results also indicated genotoxicity in the same range as the ADI values for this compound. Whether intestinal epithelial cells are more sensitive to pesticides compared to white blood cells cannot be ascertained based on the overall scarcity of comparable studies in human cell lines. Notably, the present study showed that the co-formulant piperonyl butoxide in pyrethroid-based products was observed to be genotoxic to human cell lines. Moreover, despite being considered non-genotoxic, both cyprodinil and deltamethrin were shown for the first time to be genotoxic to Caco-2 cells. Our results confirm similar previous findings related to the genotoxicity of different pesticides. However, further studies are recommended to confirm the genotoxicity of pesticides and mixtures that have been shown for the first time to be genotoxic in the present work or where no clear dose-response was observed. This study was successful in increasing the scientific evidence regarding the genotoxicity effects of commonly used pesticides, highlighting the need for further research on human cells and incorporating lower-dose concentration ranges in risk assessment studies. This study also confirms the validity of a component-based approach in genotoxicity assessment of pesticide mixtures.

## Data Availability

No datasets were generated or analyzed during the current study.
